# COVID-19 Vaccines and Axillary Nerve Dysfunction: A Case Report

**DOI:** 10.7759/cureus.49269

**Published:** 2023-11-22

**Authors:** Andrea McCarthy, Barry O'Neill

**Affiliations:** 1 Trauma and Orthopedic Surgery, St. James’s Hospital, Dublin, IRL; 2 Orthopedics, Sligo University Hospital, Sligo, IRL

**Keywords:** trauma and orthopedics, covid-19 vaccine complication, covid 19, deltoid muscle, axillary nerve injury

## Abstract

COVID-19 resulted in a worldwide pandemic and the rapid introduction of vaccines in an attempt to mitigate it. Neuritis and neuropathy after intramuscular injection had been previously seen with influenza vaccines and appear to be a side effect of the COVID-19 vaccine as well. In the following report, we present the case of a 43-year-old female who developed axillary nerve symptoms after administration of the COVID-19 vaccine and her subsequent recovery.

## Introduction

In December 2019, a novel coronavirus causing a series of severe and atypical respiratory symptoms was discovered in Wuhan in the Hubei Province of China [1]. The disease caused by this virus, now named SARS-CoV-2 (COVID-19), resulted in a worldwide pandemic causing considerable morbidity and mortality as well as worsening economic hardship globally [2].

Initial treatment of the disease revolved around preventive and supportive measures. However, the introduction of a vaccine provided hope of mitigating this. Vaccines, like all other medical procedures and medications, are, however, not without risk. Neuropathy and Brachial Plexus neuritis, while a rare complication of vaccine instrumentation, is still possible [3]. Historically, when these cases have been reported in the literature, they have been associated with the influenza vaccine or occur in developing countries [3]. We present this rare case of axillary nerve injury post COVID-19 booster vaccine.

## Case presentation

We present the case of a 43-year-old right-hand dominant care assistant who had multiple presentations to the emergency department (ED) after receiving for COVID-19 vaccine booster (Moderna). The patient received her vaccine booster on January 23, 2022 and on January 24, 2022 awoke with severe pain in the left arm with radiation to the scapular area. There was no other preceding trauma prior to the booster vaccine. The patient denied fevers at her initial presentation to the ED on January 30, 2022 but reported not being able to sleep secondary to the pain. She denied any bowel or bladder symptoms. The patient denied any history of cardiac issues.

**Figure 1:**
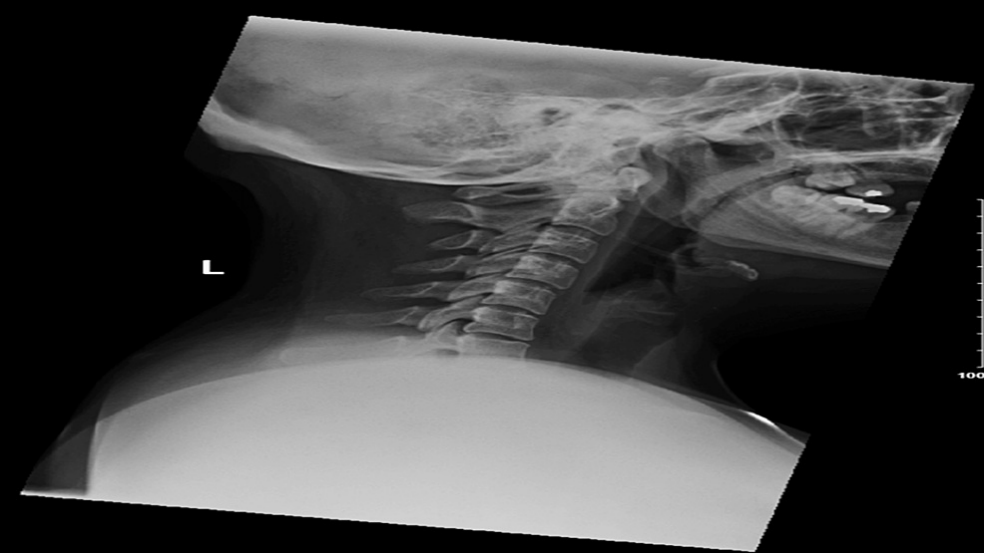
Lateral view of cervical spine taken on x-ray for the patient X-ray taken at the initial presentation of the patient to the emergency department at the onset of symptoms; no evidence of fractures or gross abnormalities to be noted.

The patient had no past medical or surgical history and was a non-smoker. Clinical examination in the ED on January 30, 2022 demonstrated no cervical spine (C-spine) tenderness and no reduced C-spine range of motion (ROM). Her hemoglobin, white cells, and inflammatory markers were all within normal range. Her D-dimer was also normal (Table 1). X-ray of the C-spine showed mild degenerative changes (Figure 1).

**Table 1:** Normal blood values and patient’s bloods at initial presentation g/dL = grams per deciliter, L = liter, mg/L = milligrams per liter, ng/mL = nanograms per milliliter

Blood	Normal Lab Values	Patient Values
Haemoglobin	11.5 – 16.5 g/dL	12.3 g/dL
White Cell Count	4-11 x 10^9^/L	6.84 x 10^9^/L
C- Reactive Protein	< 5 mg/L	3 mg/L
D-Dimer	< 500 ng/mL	<100 ng/mL

She was sent to physiotherapy and booked into ED review clinic for February 7, 2022. The patient was told that if anything changed in the meantime, she should return to the ED.

On February 5, 2022, she returned to the ED, complaining of worsening pain despite significant analgesia which she was taking regularly (Keral, Diazepam, and Tramadol). She once again denied any preceding history other than the receipt of her booster vaccine and denied sustaining any injury between presentations. The patient reported continued radiation of pain to the left scapula and on examination, the patient had full passive ROM of the shoulder joint but reduced active movements secondary to pain. Her sensation was subjectively reduced in the left upper limb in the C5-C7 distribution.

**Figure 2:**
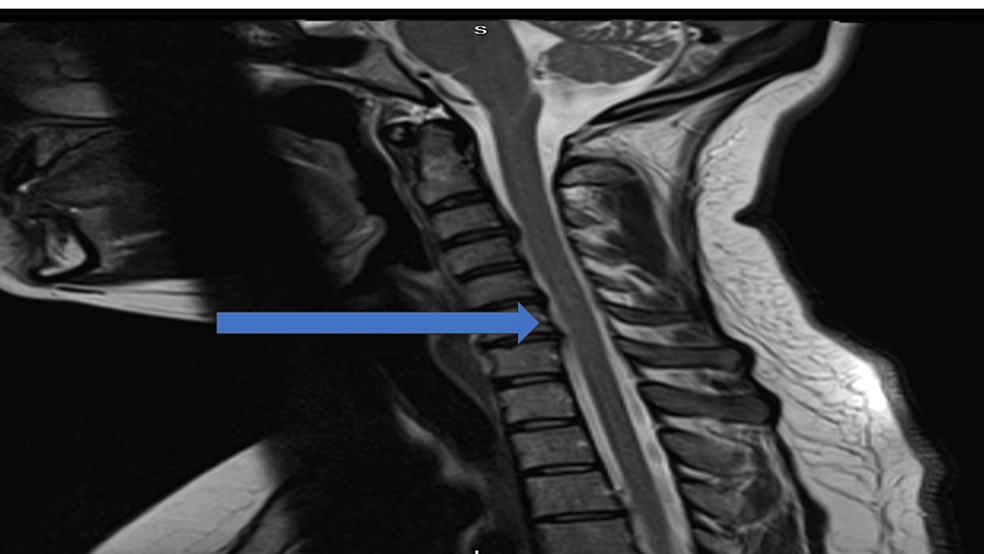
Sagittal view of the cervical spine on magnetic resonance imaging for the patient Above is a sagittal cut from the cervical spine MRI of the patient in the case report. The area marked with the arrow demonstrates the area where the patient nerve roots for the affected area would disperse from. The view also shows that there is no significant compression at this level which could cause the patient's symptoms.

She returned to the ED review clinic on February 7, 2022, and as her symptoms had failed to improve, she was referred to orthopedics for review. The orthopedic team on call examined her and found her to have reduced sensation in the C5-6 distribution and her power was reduced on the abduction of the affected upper limb. Her reflexes were normal. She was tender on palpation over her left shoulder and medial scapular region. The patient was started on gabapentin, booked for an outpatient MRI, and booked into the orthopedic clinic for further follow-up and review (Figure 2).

The patient was reviewed in the Orthopedic Trauma clinic on February 14, 2022; she reported some improvement in her pain since the commencement of the Gabapentin but also demonstrated weakness in the abduction of the affected arm. Her Deltoid muscle, where she had received the booster COVID-19 vaccine was tender to palpation. Her neurological examination was otherwise unremarkable at the orthopedic clinic with normal tone and reflexes and no evidence of fasciculations. The patient was reviewed by the orthopedic consultant who suspected an axillary nerve chemical neuritis induced by the COVID-19 booster and sent the patient for nerve conduction studies for further assessment.

The patient attended nerve conduction studies on April 6, 2022 which showed a low-grade axillary neuropathy with evidence of ongoing recovery. Her MRI scan demonstrated no significant findings. She was reviewed again in the orthopedic trauma clinic on was May 16, 2022, where the patient reported improvement of her symptoms, with lessening pain and evidence of initial improvement in the function of her arm.

The patient had follow-up nerve conduction studies in September 2022 and March 2023 respectively. The studies conducted in September 2022 showed an amplitude response of 6mV (Table 2). However, her deltoid function on needle EMG showed evidence of good recovery with an 80%-85% normal recruitment pattern. Her repeated studies in March 2023 showed an improved and near normal amplitude response of 9.2 mV and deltoid function had also improved to 95% normal recruitment pattern (Table 2). She was reviewed in the clinic in June 2023 where she reported full resolution of her pain and intermittent paresthesia. Clinically, her arm abduction was 5/5 power and equal to the opposite arm.

**Table 2:** Normative values for axillary nerve on nerve conduction studies and findings for each set of nerve conduction studies for patient Mv = Millivolts

Parameters:	Normal Values:	Nerve Conduction Studies 1	Nerve Conduction Studies 2	Nerve Conduction Studies 3
Amplitude	10Mv	4Mv	6Mv	9.2Mv
Recruitment pattern	95%	50%	80-85%	95%

## Discussion

The recommended site for the administration of vaccines and other intramuscular medications in adults is the deltoid muscle, and generally, vaccines administered here are well tolerated with minimal side effects [4]. However, significant peripheral nerve injuries may occur and have previously been documented in the literature associated with the influenza vaccine; this may be due to direct trauma from the vaccine needle, hematoma formation, and nerve compression or chemical neuritis secondary to the contents of the vaccine [5].

While nerve injuries and neuritis are considered to be a rare side effects of vaccines, it is worth noting that Moderna, the brand of vaccine the patient received, has been listed in the literature in several case reports in association with transverse myelitis, small nerve neuropathy, and neuritis, especially optic neuritis, after administration. However, a review of the ingredients that constitute Moderna, did not yield any particular compound which is noxious to nerves.

The development of the patient’s presentation soon after the booster vaccination and the exclusion of other known etiologies support the possible causal association between the patient’s nerve conduction studies confirmed neuropathy and her COVID-19 booster injection. Several similar case reports, with similar clinical histories and presentations, have been reported in the literature.

In the literature, however, there is some variation between the injection of the vaccine and onset of symptoms with other studies quoting between four and 18 days. Sharma et al. reported a similar case of axillary nerve injury after the COVID-19 vaccine, with symptoms starting within one day of administration like our patient. However, in their report, the vaccine used was Covishield while in our case, Moderna was the vaccine administered [4].

Our patient’s symptoms began to improve when treated with gabapentin; this is consistent with the case report published by Waheed et al. [6], though their case occurred with the Pfizer vaccine while ours occurred with Moderna. While our patient’s symptoms are now improved, there have been a considerable number of months where her ability to financially support herself and complete basic activities of daily living have been affected.

## Conclusions

While vaccination has considerable benefits for the individual and the community in the wake of the COVID-19 pandemic, it must be considered that they, like all medical procedures, are not without risk. While the deltoid is a generally safe place for vaccine administration and complications of vaccination are rare, neuropathy can be devastating for the patient, affecting their ability to function and take care of basic everyday tasks. Thus, it is important that considerable effort is put into identifying the correct anatomy and training people on how to appropriately give these vaccinations.
